# Therapeutic Targets in the Management of Dry Eye Disease Associated with Sjögren’s Syndrome: An Updated Review of Current Insights and Future Perspectives

**DOI:** 10.3390/jcm13061777

**Published:** 2024-03-20

**Authors:** Abdulmohsen Almulhim

**Affiliations:** Department of Ophthalmology, College of Medicine, Jouf University, Sakaka 72388, Saudi Arabia; akalmulhim@ju.edu.sa; Tel.: +966-507788885

**Keywords:** Sjögren’s syndrome, dry eye disease, management, therapy, topical therapy

## Abstract

Dry eye disease (DED) is a continuing medical challenge, further worsened in the autoimmune inflammatory hyperactivation milieu of Sjögren’s syndrome (SS) due to disturbances to innate and adaptive immunity with malfunctioning neuro-endocrine control. However, the pathogenetic mechanisms of SS DED are not fully established. This review summarized the available evidence, from systematic reviews, meta-analyses, and randomized clinical trials, for the efficacy and safety of the available ocular therapeutics for the management of SS DED. Relevant studies were obtained from major databases using appropriate keywords. The available largely empirical symptomatic, supportive, and restorative treatments have significant limitations as they do not alter local and systemic disease progression. Topical therapies have expanded to include biologics, surgical approaches, scleral lens fitting, the management of lid margin disease, systemic treatments, nutritional support, and the transplantation of stem cells. They are not curative, as they cannot permanently restore the ocular surface’s homeostasis. These approaches are efficacious in the short term in most studies, with more significant variability in outcome measures among studies in the long term. This review offers an interdisciplinary perspective that enriches our understanding of SS DED. This updated review addresses current knowledge gaps and identifies promising areas for future research to overcome this medical challenge.

## 1. Introduction

Sjögren’s syndrome (SS) is a common diffuse connective tissue autoimmune exocrinopathy with widespread systemic extraglandular effects involving the lungs, kidneys, joints, skin, thyroid, gastrointestinal tract, including the liver and pancreas, and cardiovascular and nervous systems, seen upon the progression of the disease [1,2]. SS has a female preponderance and occurs as a primary disease or in association with other connective tissue autoimmune disorders and diseases such as rheumatoid arthritis and systemic lupus erythematosus. SS is also associated with human immunodeficiency syndrome, hepatitis C and COVID-19 infection, chronic graft-versus-host disease after allogeneic bone marrow transplantation, neoplastic and myeloblastic syndromes, chronic biliary cirrhosis, fibromyalgia, and chronic fatigue syndrome. Predictive models significantly associate SS with preceding female hormone replacement therapy, fibromyalgia, lymphoma, body mass index, osteoporosis, and diabetes [3,4,5,6]. The precise pathogenic mechanisms of SS remain incompletely understood. This hinders the proper management of the syndrome, which is mainly symptomatic. Inflammation induced by lymphocytic infiltration (specifically CD4+ T and B cells, and plasma cells) of the salivary, lacrimal, nasal, and sebaceous glands or vaginal mucosa (acinar and ductal cells) and other tissues is a pathognomonic hallmark, with progressive damage to these tissues and reduced secretions, culminating in severe dryness (Sicca syndrome; xerophthalmia, xerostomia, and xeroderma) and systemic symptoms [2,4]. Xerophthalmia is unrelated to Sjögren’s syndrome and is induced by vitamin A deficiency, although vitamin A and vitamin D deficiencies have been linked with the syndrome. The constellation of autoantibodies (particularly antinuclear anti-Ro/SS-A and anti-La/SS-B) indicates a loss of B cell tolerance along with dysregulated activity in the interleukin (IL)-1 system. The disease has detrimental clinical, quality-of-life, and financial burdens on patients [7,8,9,10]. 

There are no well-controlled randomized trials for treating SS, and treatment is mainly empirical—symptomatic, supportive, and restorative/replacement [11,12]. Studies tend to focus mainly on xerostomia and the salivary glands because they are so easy to access and most affected [6,13]. At present, there are no permanent treatment options for SS. Presently, widely utilized therapies include symptomatic management and replacement therapy to alleviate the symptoms experienced by patients, reduce disease progression, and improve quality of life [7,14,15]. The various medications employed in SS treatment can be categorized into local, systemic, and biological therapies. Recently, the first-ever US clinical practice guidelines (CPG) for Sjögren’s syndrome were developed under the auspices of the Sjögren’s Syndrome Foundation for treating the oral, ocular, and rheumatologic/systemic manifestations [16,17]. The ocular guidelines stressed the necessity for a thorough evaluation of dry eye disease (DED) by a cornea specialist or eye care provider to determine the mechanism of dryness in SS patients (i.e., aqueous-deficient dry eye (ADDE) vs. evaporative dry eye (EDE)) and level of severity. SS DED is commonly characterized by ADDE. Nonetheless, recent works of literature have reported that EDE also can occur in SS due to meibomian gland dysfunction (MGD) [18,19]. DED associated with SS presents a significant clinical challenge, impacting patient comfort and vision. Therefore, given recent advancements in research, there is a need to consolidate the current understanding and identify effective therapeutic targets for optimal management, which can assist clinicians in enhancing patient outcomes and quality of life through informed treatment decisions. Furthermore, identifying existing knowledge gaps in this field will provide insights for future research directions. This review aims to highlight the current ocular therapeutics for SS and emphasize ocular treatment options that target different mediators of SS DED pathogenesis and the role of systemic management. Hence, the unmet needs of managing DED associated with SS can be identified. Furthermore, this review identified existing knowledge gaps that provide insights for future research directions.

## 2. Methods and Search Strategies

To ensure that all good-quality articles are accounted for, all-inclusive search criteria were developed. The author included systematic reviews, meta-analyses, and randomized clinical trials in English in the past ten years and excluded case studies, case reports, non-English manuscripts, and grey literature. Furthermore, the author excluded articles for which the full text was not available. A flowchart detailing the various steps of the search process, including the number of documents obtained at each step and the documents eliminated based on the application of different search criteria, is given as Appendix A. The necessary articles for this review were obtained from the Cochrane Library (contains the DARE database), Embase, the National Library of Medicine (Medline—PubMed), the Center for Evidence-Based Medicine and Scopus, Web of Science, ClinicalTrials.gov (18 July 2023), and the World Health Organization (WHO) International Clinical Trials Registry Platform (ICTRP). These online sources were searched for eligible articles that were aligned with the purpose of this review. Search terms were Sjögren’s syndrome (capital or lower-case S); Treatment (capital or lower-case T); Therapy (capital or lower-case T); management; and dry eye, once as a MeSH term and once as a free text word. 

## 3. Pathogenetic Mechanisms of SS and Associated DED

The etiopathogenesis of disease remains obscure amid a scenario of autoreactive B and T cells induced by (1) genetic predisposition [e.g., polymorphisms of human leukocyte antigen (HLA)-DR and HLA-DQ gene regions that elicit the production of different autoantibodies], (2) epigenetic susceptibility (abnormalities related to microRNA expression, DNA methylation, and histone acetylation), (3) hormonal changes (particularly for sex hormones), and (4) environment triggers (viral and bacterial infections and microbiota dysbiosis) [20]. Polymorphisms situated at six distinct loci are also associated with SS: IRF5, STAT4, BLK, IL12A, TNIP1, and CXCR5 [21,22]. These polymorphic loci are implicated in: (1) interferon activation of the innate immune system, (2) B cell recruitment to lymphoid follicles and activation (through CXCR5, B cell receptor, and BLK), and (3) T cell activation due to HLA susceptibility via the IL-12-IFN-γ pathway [23]. The abnormally recruited and retained lymphocytes (dendritic cells, T cells, and B cells) in the tissues are subsequently stimulated by the proinflammatory cytokines (IL-1β, IFN- α/γ, TNF-α, and B cell-activating factors) secreted by these lymphocytes and epithelial cells. The chronic stimulation of apoptosis-resistant B cells’ maturation into self-reactive B cells locally produces autoantibodies. This establishes a mutagenic lymphomagenesis germinal center-like structure that progresses to lymphoma in severe cases [20,24]. As surrogate markers of disease severity, there are elevated concentrations of IL-1β, IL-2, IL-4, IL-6, IL-9, IL-12, IL-17A, IFN-γ, and TNF-α cytokines, and CCL2, CCL3, CCL4, and CXCL8 chemokines. The levels of MMP-9, FGF, VEGF-A, IL1RA, sICAM-1, and sTNFR1 soluble receptors, NGF, substance P, and serotonin neurotrophic factors were also increased, while the concentrations of IL-7, IL-17F, CXCL1, CXCL10, EGF, and lactoferrin were reduced, in DED patients compared with healthy controls [25,26]. 

The clinical consequences of Sicca syndrome can negatively affect patients’ quality of life, as diminished tear production generally leads to eye irritation, itching, burning, and destruction of the conjunctival and corneal epithelium (keratoconjunctivitis Sicca). This leads to symptoms such as the sensation of a foreign body, burning, itching, eyestrain, sensitivity to light, blurred vision, and redness of the eyes. These symptoms worsen with extended periods of visual tasks and environmental factors such as low humidity and extreme cold. There is a > 9:1 female-to-male ratio for individuals aged ≥50 for SS, a prevalence of up to 3% of the population, and a more severe presentation in females than males [20,27]. In its secondary form, SS is associated with autoimmune or rheumatic disorders, including rheumatoid arthritis (up to 50% of patients), lupus erythematosus (up to 25% of patients), scleroderma, autoimmune thyroiditis, multiple sclerosis, celiac disease, spondylo-arthropathy, and fibromyalgia, and several malignancies, principally non-Hodgkin lymphoma [28]. There is often confusion and misinterpretation regarding the clinical manifestations of SS. They can be mistaken for symptoms of other medical conditions or iatrogenic disorders. To discover new potential biomarkers and targets for therapy, tear proteome analysis showed that the tear content of apolipoprotein D, S100A6, S100A8, and ceruloplasmin differed the most between DED and healthy controls. Simultaneously, antileukoproteinase, phospholipase A2, and lactoperoxidase levels permitted differentiation among meibomian gland dysfunction (MGD) and DED patients, and the alterations in the levels of annexin A1, clusterin, and α1-acid glycoprotein 1 differentiated MGD patients from healthy controls [29]. Analyzing feces and plasma metabolomics in primary SS patients vs. controls showed that 9-OxoODE, 2-hydroxyethanesulfonate, diepoxyoctadecanoate, and tyramine are metabolism-related diagnostic markers [30]. The SS tear contents of S100A6, MMP-9, and CST4 differentiated patients from healthy controls [31]. 

## 4. General Management of DED Associated with SS

Along with the management of the systemic manifestations of SS, the treatment strategies for associated DED have evolved over the past two decades, but numerous principles remain unchanged. There is no cure or specific treatment for SS that can permanently restore the secretion of exocrine glands and ocular surface homeostasis. Therefore, treatment is generally symptomatic, supportive, and restorative. Primarily, the exchange of information between the treating physician and patient is vital to ensuring individuals with SS DED recognize the nature of the DED, exacerbating factors, and treatment approaches. Due to the various underlying factors contributing to DED, treatment approaches have broadened to encompass nutritional support and the management of lid-margin disease, immunosuppressive biologics, stem cells, and topical therapy. A step-by-step approach to the management of SS DED is advised. All these management approaches are aimed at restoring the ocular surface’s homeostasis by interrupting the detrimental cycle of DED and providing sustainable measures to prevent its recurrence [32,33,34]. 

Standard treatment for EDE (i.e., MGD), also one of the prevalent conditions in SS patients, encompasses eyelid hygiene and warming. It is important to note that SS-related DED primarily involves tear deficiency, with secondary occurrences of MGD [19,35]. Even though artificial tears are extensively utilized globally and come in various formulations, there is a scarcity of studies that directly compare different types of artificial tears to determine their superiority [36]. Almost all artificial tears have an aqueous base but may vary in osmolarity, viscosity, pH, and composite viscosity-enhancing agents. The hydrogels that have been used in artificial tear substitutes include hydroxypropyl methylcellulose, carboxymethylcellulose, polyvinyl alcohol, carbopol, polyvinylpyrrolidone, polyethylene glycol, dextran, hyaluronic acid, or carbomer 940 (polyacrylic acid) and hydroxypropyl methylcellulose [37,38]. 

### 4.1. Autologous Serum Eye Drops

Randomized clinical trials (RCTs) showed that autologous/allogeneic serum eye drops provide lubrication that closely mimics natural tears related to the biochemical components, and they gained popularity as a safe, well-tolerated, and effective second-line therapy for DED, at least in the short term [39]. The variability and low-certainty evidence among the reported RCTs for serum eye drops may be attributed to the fact that SS patients have systemic inflammatory and pro-oxidant status due to the hyperactivated immune reaction. They yield some sort of serum proinflammatory cytokine storm that would have detrimental local effects, at least in long-term scenarios. Therefore, donor healthy allogeneic serum is expected to be more effective [39,40,41]. Autologous serum eye drops are proven to be safe and effective for managing SS DED. Hwang J et al. [42] and Liu Y et al. [43] demonstrated the effectiveness of autologous serum among DED SS patients. However, Hwang J et al. [42] questioned their effectiveness in managing DED linked with secondary SS due to increased serum proinflammatory cytokine levels.

### 4.2. Carbopol 940 in Gel

Carbopol is a specific type of carbomer. Many commercial eye drops contain Carbopol 940 or other derived polymers to achieve better corneal retention and higher bioavailability. Carbopol 940 is commonly applied as a gelling agent in gel formulations. It is an acrylic polymer, is both non-toxic and non-irritating to the eyes, and is one of the most widely utilized polymers in ophthalmic formulations [44]. Furthermore, recent evidence indicates that Carbopol 940 does not cause corneal nerve damage and is relatively safe to use among patients with DED [44]. A study by Chung et al. among 412 patients reported that Carbomer-based formulations improved the vision issues among DED patients [45], and the same findings are supported by Caretti L et al. [46]. Moreover, under physiological pH conditions, carbopol interacts with mucus and biological surfaces by forming hydrogen bonds with ionized carbonyl functionalities, forming a strengthened gel lattice [47].

### 4.3. Hyaluronic Acid

Hyaluronic acid (HA) is a natural and high-molecular-weight polysaccharide. HA is extensively present throughout the extracellular matrix of connective tissue [48]. Its physio-chemical properties, such as highly anionic properties, have the ability to attract water, causing swelling and volumizing effects, thereby offering structural support. Hence, HA is often used in several clinical practice specialties, including dermatology, orthopedics, and ophthalmology [48]. For the first time in artificial eye drops, high-molecular-weight HA (HM-HA) eye drops were proven to be an acceptable substitute for autologous serum eye drops [49]. Some individuals with DED viewed HM-HA drops as better than patient serum or other tested tear substitutes. Ideally, HA-based artificial tears must comprise MW-HA with a low polydispersion index and increased viscosity at a lesser shear rate (without exceeding the blur threshold), as confirmed by a recent investigation [50]. A study by Aragona P et al. [51] stated that treatment with sodium hyaluronate should be considered for managing SS DED. A recent study by Fogagnolo P et al. [52] reported that HA and ginkgo biloba eye drops have the potential to decrease DED signs and symptoms among patients who have undergone cataract operations. Furthermore, it is well-tolerated by patients and highly safe. 

## 5. Medicaments in the Management of DED

Topical drugs that decrease ocular surface inflammation and are often utilized in DED include glucocorticoids, cyclosporin, and, sometimes, tacrolimus. Topical steroids, like dexamethasone, methylprednisolone, and fluorometholone eye drops, act by decreasing inflammatory cytokine expression. Several works of literature, including RCTs, have demonstrated improved ocular surface parameters after short-term use in DED patients [38]. Targeted biologic therapeutic monoclonal antibodies and agents work to deplete systemic B cells (e.g., rituximab), inhibit local B cell activation (e.g., belimumab and ianalumab), and interfere with co-signaling molecules (e.g., anti-CD40 iscalimab) or proinflammatory cytokines (e.g., IL-6 tocilizumab) and small-molecule inhibitors of their signaling pathways. These immunosuppressive biologics had variable efficacy outcomes in improving salivary and ocular activity scores, which may be attributed to (1) their inefficacy, (2) patients with irreversible exocrine glandular failure at advanced disease stages, (3) the small sample size studied and high heterogeneity among SS disease presentations, and/or, (4) inappropriate recruitment strategies in clinical trials, and the outcome measures used [53,54,55,56].

### 5.1. Topical Corticosteroids

The benefits of topical corticosteroids such as dexamethasone, methylprednisolone, and fluorometholone have been evaluated by several authors [57,58]. They have several limitations, including increased pressure in the eyeball, cataract formation, and infections in severe DED. The use of these drugs is usually limited to a short period (2–4 weeks) [20,59]. A recent study by Ryu K et al. [60] reported that topical glucocorticoids had significant benefits among patients with refractory DED. Nonetheless, corticosteroids play some crucial roles in the clinical management of SS DED patients. Moreover, different types of corticosteroids varied in their potency, duration of action, safety profile, used combinations, and patient tolerability [58,61,62]. Regarding potency and efficacy, dexamethasone and prednisolone are considered highly potent. However, their safety profile is lower than that of other types of corticosteroids (fluorometholone and loteprednol etabonate), especially when used long term, as SS is a life-long condition [57,61,62]. On evaluating the efficacy of fluorometholone in SS DED patients, a comparative study by Lin, T., and Gong, L. [58] stated that fluorometholone was quicker in improving DED-related symptoms in SS, and fluorometholone was generally well tolerated. A recent study by Fondi K et al. [63] demonstrated that eye drops with a combination of hydrocortisone and ciclosporin A improved patients‘ symptoms and might be beneficial for long-term and improved vision quality among SS DED patients. In addition, patients’ tolerability is critical in choosing the type of corticosteroid to be used in managing DED symptoms [58,64].

### 5.2. Topical Cyclosporine

Cyclosporine affects both cellular and humoral immune responses by inhibiting calcineurin-dependent T cell events, thereby inhibiting the release of proinflammatory cytokines and inflammation, which disrupt tear secretion [65]. Topical cyclosporine comes in 0.05%, 0.1%, and 0.09% concentrations, and the therapeutic effect usually takes weeks to manifest when applied twice daily. With topical cyclosporine therapy, improvements in clinical outcomes are also seen in the immunohistochemistry of the conjunctiva, such as decreased levels of activated T-lymphocytes and apoptotic cells [66,67]. In 2018, the US FDA approved Cequa^®^ (Sun Pharmaceutical Industries Ltd, Mumbai, India), a 0.09% cyclosporine ophthalmic solution for managing DED and keratoconjunctivitis Sicca. This is a preservative-free and nanomicellar topical formulation that has been proven to deliver the required concentration of Cyclosporine to the ocular tissue. Furthermore, Cequa^®^ offers a well-tolerated and effective treatment option for individuals suffering from DED and keratoconjunctivitis Sicca [68,69]. However, Cequa^®^ has not been approved by the US FDA as yet for managing SS DED, as the clinical trial (NCT04835623) is ongoing and awaiting the final outcome [70]. Another US FDA-approved cyclosporin formulation is Restasis^®^ (Allergan, Inc., Irvine, CA, USA) [a 0.05% cyclosporine ophthalmic emulsion]. However, there is no unanimous agreement regarding its efficacy [66,71].

### 5.3. Other Novel Drug-Delivery Systems (DDSs) for the Management of SS DED

Several DDSs are used for ophthalmic topical medications, and novel DDSs are emerging. These DDSs are used in all DEDs, including SS DED. In addition to the earlier-mentioned DDSs, the novel DDSs are nanoparticles, liposomes, dendrimers, contact lenses, emulsions (micro and macro), and niosomes. The purposes of DDSs are to reduce the side effects, as the therapeutic agents are specifically attracted to the ocular tissue, increase bioavailability, and improve adherence by the patients. Each DDS offers unique advantages regarding drug release kinetics, bioavailability, and visual surface compatibility [72,73,74]. The nanoparticles utilized in ophthalmic DDSs are generally composed of lipids, including fatty acids and triglycerides, combined with natural or synthetic polymers. Liposomes act by improving the penetration of drug components by binding to the ocular surface and enhancing attaching time [74,75]. 

### 5.4. Anti-Inflammatory Polyunsaturated Fatty Acids and Topical N-Acetylcysteine

Essential polyunsaturated fatty acids, especially omega-3 fatty acids, have also been evaluated for the treatment of DED due to their anti-inflammatory properties [76]. However, the human body cannot produce these nutrients, so they have to be acquired from dietary sources. Nonetheless, taking more than 3 mg of omega-3 fatty acids per day is not recommended. Some patients may experience excessive bleeding after receiving a high dose. Moreover, inflammatory cascades are triggered by omega-6 essential fatty acids, which are precursors to eicosanoids such as prostaglandins, thromboxanes, and leukotrienes. Omega-3 fatty acids (e.g., eicosapentaenoic acid and docosahexaenoic acid) competitively inhibit cyclooxygenases and 5-lipoxygenase activity that otherwise would produce proinflammatory eicosanoids with arachidonate as the substrate. Consequently, omega-3 fatty acids are believed to promote an anti-inflammatory physiologic state in the body [77]. The effectiveness of anti-inflammatory omega-3 polyunsaturated fatty acid supplementation in the treatment of DE syndrome has been shown in multiple studies [6,78]. Omega-3 dietary supplements have an additive effect on the tear film indices of patients with DE syndrome after phacoemulsification [79]. Topical N-acetylcysteine, in its simple and nanoparticle chitosan-N-acetylcysteine (Lacrimera^®^ [CROMA-PHARMA GmbH, Leobendorf, Austria]) forms, has proven safe and effective in the treatment of surface ocular pathology, including DED, particularly for low-dose preparations. N-acetylcysteine is a precursor to generating glutathione as a significant antioxidant and is a direct antioxidant and anti-inflammatory [80,81,82].

### 5.5. Lifitegrast (Xiidra^®^ [Bausch + Lomb U.S, Bridgewater, NJUSA])

This small-molecule integrin antagonist, approved by the FDA in 2016, acts by inhibiting lymphocyte function-associated antigen-1 (LFA-1), a cell adhesion molecule found on the surface of lymphocytes. Hence, it reduces ocular surface inflammation associated with DED by blocking the interaction between lymphocytes and the ocular surface, thereby reducing the symptoms of dry eye disease. Numerous recent pieces of evidence, including RCTs, have demonstrated that lifitegrast is an influential novel medicine for reducing the signs and symptoms of DED with a high safety profile [83,84,85]. On evaluating the effectiveness of lifitegrast (Xiidra^®^) among 600 individuals with DED in the US and Canada, Hovanesian JA et al. [86] reported that lifitegrast reduced treatment burden and improved quality of life among the patients with DED.

## 6. Off-Label Therapeutic Management 

In DED, systemic medications such as pilocarpine and cevimeline are not usually prescribed because of their side effects (excessive sweating and nausea) and lack of effectiveness in reducing ocular dryness. The agents are approved by the FDA for managing oral dryness among SS patients. On evaluating the effectiveness of oral pilocarpine, specifically among patients with severe DED, Kawakita T et al. [87] demonstrated a beneficial role in those patients who were refractory to conventional agents. Another study by Felberg S et al. [88] in 2022 evaluated the use of oral pilocarpine to improve the quality of life of SS DED patients. They demonstrated a greater improvement in vision-related quality of life among oral pilocarpine-treated patients than the comparison group. However, they observed a higher rate of side effects such as sweating and excessive salivation. A 3% solution of diquafosol is available for ophthalmic use. Specifically, it reduces DE symptoms by stimulating the secretion of water and mucin through its action on the PY2Y2 receptors on the conjunctiva epithelium and goblet cells. There has been evidence of improvement in DE symptoms in SS patients [7]. One of the proposed mechanisms by which spironolactone may improve DED symptoms is through its anti-androgenic properties and restoration of corneal integrity, as explored by Rodrigues-Braz D et al. [89] in 2023 and other authors [90]. In 2023, Wong CW et al. [91] evaluated the off-label use of spironolactone in managing DED. They found that topical spironolactone could enhance tear film content and control the inflammatory processes linked with DED. Light-based therapies are novel and another off-label way of managing DED. There are two kinds of light-based therapies: intense pulsed light and low-level light therapy. Currently, the FDA approves light-based therapy for dermatological management, and this method acts by lowering the inflammation linked to EDE and decreasing microorganism growth [92]. Considering the wound healing tissue regeneration mechanism, platelet-rich plasma (PRP) treatment is commonly used in managing orthopedics and dermatology specialties. However, the use of PRP in DED is still limited. Nonetheless, the results of a preliminary study from Canada by Murtaza F et al. [93] provided promising evidence for the benefit of PEP in DED patients. In 2024, Kwaku Akowuah P et al. [94] further explored the use of PRP in DED patients to reduce symptoms and enhance patients’ quality of life. Regarding antibiotics, the FDA approved using them for some specific indications. Nonetheless, they are considered off-label if the antibiotic is solely used for its anti-inflammatory properties (such as doxycycline or macrolide) [95,96]. 

## 7. Surgical Treatment, Scleral Lens Fitting, and Stem Cell Therapy

In patients with more severe DE, the use of goggles increases local humidity, as do punctal plugs, punctal occlusion, and partial or total tarsorrhaphy, which are beneficial as they retain tears on the ocular surface for a longer time [7,97,98]. Contact lenses play an important role in managing DED. Two types of contact lenses are currently commonly used: soft scleral lenses and rigid gas permeable (RGP) lenses. Generally, RGP lenses are preferred over soft lenses in managing DED due to their superior durability and oxygen penetrability [99,100]. The fitting of a contact lens that is simple, fenestrated, and oxygen-permeable has a range of therapeutic, ocular surface disease-mitigating, and optical applications. They protect against environmental causes and mechanical injuries induced by deformed eyelids, uphold surface moisture and tear balance, and provide consistent drug administration to the eye’s surface. Ocular surface diseases, including DED, showed significant improvements in terms of DED symptoms and quality of life [99,101]. However, complications related to scleral lens wear include infection, insertion and removal obstacles, and anomalies like conjunctival prolapse and epithelial swelling. These also include rare hypoxic and inflammatory complications [102,103,104,105]. Novel surgical procedures also include transposition or transplantation of the salivary glands, amniotic membrane transplantation, and stem cell-based injections into the lacrimal gland [106]. The transplantation of mesenchymal and allogeneic hematopoietic stem cells, with multi-potentiality, trophic, and immunomodulatory properties, is under scrutiny, with variable promising responses and limitations, due mainly to graft rejection despite local immunosuppression [107,108,109,110]. As DEDs in general, those associated with SS, in particular, are still medically unsolved problems, curative treatment is necessary, and the investigation pipeline is full of promising new approaches registered as clinical trials. Bee honey products are among the promising new topical therapies [111,112,113,114].

## 8. Systemic Management Strategies of DED Associated with SS

Even though DED can occur in other systematic conditions, the severity of DED is significantly higher among patients suffering from SS, as determined by Yu K et al. [60] through an RCT. According to the European Alliance of Associations for Rheumatology, in general, DED treatment should begin with local therapy, and systemic treatment should be given in active cases of SS [34]. Systemic management aims to address the underlying autoimmune process and reduce its effects on the ocular surface by suppressing the abnormal immune response associated with SS. The pharmacological treatment recommended is corticosteroids, followed by immunomodulators and disease-modifying antirheumatic drugs (DMARDs). Moreover, the systemic nature of this disease could give an opportunity to educate patients on the importance of adhering to the medication prescribed by the treating physician, which, in turn, would improve DED outcomes [34,115].

## 9. Knowledge Gaps, Challenges, and Future Research Directions for DED Associated with SS

Despite the significant innovations that research communities have achieved in evaluating various aspects of treatment modalities for DED SS, this review explored several complexities and challenges that still need to be addressed, leading to difficulty handling such cases. These challenges and knowledge gaps are summarized below and can serve as catalysts for future research directions aimed at overcoming this medical challenge.

1. The accurate mechanism and pathology behind DED associated with SS are still uncovered. Hence, establishing a deeper understanding of interconnecting factors such as immune dysregulation, inflammatory processes, and ocular surface changes is critical to enabling targeted therapies to be planned by treating ophthalmologists.
2. The early detection and monitoring of DED SS remains an obstacle, leading to challenges in timely interventions. Hence, developing and testing a non-invasive early diagnostic tool with high sensitivity and specificity is necessary. These can be achieved by identifying early diagnosis biomarkers.
3. Despite recent advancements, there is still a need for novel therapeutic targets for DED in SS. Exploring innovative approaches, such as gene therapy, stem cell therapy, and targeted immunomodulation, holds promise for future treatment modalities.
4. SS is a systemic autoimmune disorder that involves the whole body and has diverse symptoms. The need for and effectiveness of the systemic management of DED SS are still unclear.
5. The eyes are regularly exposed to hormones (such as progesterone) in the bloodstream and have receptors that are tailored to these hormones. Research exploring the management approach for DED in SS patients in the context of hormonal influences on the ocular surface is very critical as it could provide valuable insights. However, the available evidence in this area needs to be improved.
6. The ocular manifestations of patients with SS vary depending upon the severity, subtype, and response to therapy, which is an area ripe for exploration. Hence, a deeper exploration of a personalized approach could be beneficial, as there is no “one size fits all” in DED SS in terms of therapeutic targets.
7. A multidisciplinary collaborative approach involving ophthalmologists and other relevant healthcare professionals is critical to the holistic management of DED associated with SS. However, there is a lack of sufficient data from research that explores integrated approaches to addressing the complex nature of this condition.
8. Even though SS and DED are far from rare disease criteria, medical professional education on these diseases is relatively low compared to other autoimmune diseases such as rheumatoid arthritis and systemic lupus erythematous. This disparity has led to physicians’ perception that DED SS is an unimportant health issue. Hence, sufficient targeted training programs for doctors, especially at the primary care level, could help with early diagnosis and timely interventions for DED SS.

Addressing these knowledge gaps and challenges through collaborative research efforts will pave the way for an improved understanding and the diagnosis and management of DED associated with SS, ultimately enhancing patient outcomes and quality of life.

## 10. Conclusions

For patients with DED, particularly those with SS, multiple treatment options are available. However, the optimal treatment approach is still uncovered. The pathogenetic multifactorial nature of DED in association with SS, the poor correlation between symptoms and signs, and the multiplicity of treatments that have not been compared in RCTs are some of its challenges. Correlating the progress in understanding the pathogenesis of SS, its ocular aspects, and Sicca syndrome in particular, there is hope that more effective therapeutic options are on the horizon for DED. This review offers various perspectives that enrich our understanding of SS-associated DED by synthesizing evidence from the latest studies conducted in different settings. This updated review addresses current knowledge gaps and identifies promising areas for future research to overcome this medical challenge. Furthermore, a deeper exploration of a personalized approach could be beneficial, as there is no “one size fits all” in DED SS in terms of therapeutic targets.

## Figures and Tables

**Figure A1:**
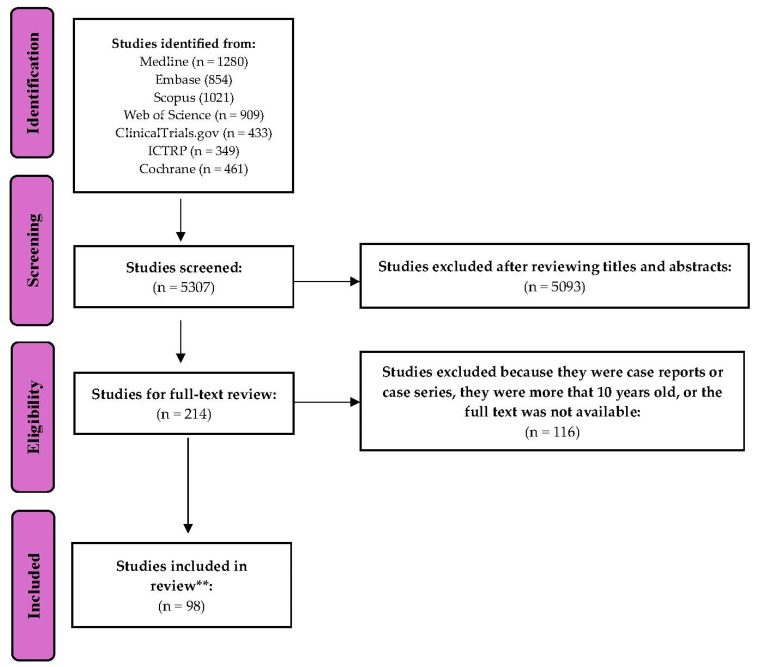
* Adapted from PRISMA (2020). ** Number of articles included in the main text.

## Data Availability

No new data was generated.
